# Iterative Development, Validation, and Certification of a Smartphone System to Assess Neonatal Jaundice: Development and Usability Study

**DOI:** 10.2196/40463

**Published:** 2023-02-28

**Authors:** Anders Aune, Gunnar Vartdal, Gabriela Jimenez Diaz, Lobke Marijn Gierman, Håkon Bergseng, Elisabeth Darj

**Affiliations:** 1 Department of Public Health and Nursing Faculty of Medicine and Health Sciences Norwegian University of Science and Technology Trondheim Norway; 2 Picterus AS Trondheim Norway; 3 Department of Clinical and Molecular Medicine Faculty of Medicine and Health Sciences Norwegian University of Science and Technology Trondheim Norway; 4 Department of Neonatology St.Olav Hospital Trondheim Norway

**Keywords:** neonatal jaundice, neonatal hyperbilirubinemia, newborns, mobile app, design, validation, regulatory processes, mobile health, mHealth, mobile phone

## Abstract

**Background:**

Medical device development is an area facing multiple challenges, resulting in a high number of products not reaching the clinical setting. Neonatal hyperbilirubinemia, manifesting as neonatal jaundice (NNJ), is an important cause of newborn morbidity and mortality. It is important to identify infants with neonatal hyperbilirubinemia at an early stage, but currently there is a lack of tools that are both accurate and affordable.

**Objective:**

This study aimed to develop a novel system to assess the presence of NNJ. The device should provide accurate results, be approved as a medical device, be easy to use, and be produced at a price that is affordable even in low-resource settings.

**Methods:**

We used an iterative approach to develop a smartphone-based system to detect the presence of NNJ. We performed technical development, followed by clinical and usability testing in parallel, after which we initiated the regulatory processes for certification. We updated the system in each iteration, and the final version underwent a clinical validation study on healthy term newborns aged 1 to 15 days before all documentation was submitted for conformity assessment to obtain Conformité Européenne (CE) certification. We developed a system that incorporates a smartphone app, a color calibration card, and a server.

**Results:**

Three iterations of the smartphone-based system were developed; the final version was approved as a medical device after complying with Medical Device Regulation guidelines. A total of 201 infants were included in the validation study. Bilirubin values using the system highly correlated with total serum or plasma bilirubin levels (*r*=0.84). The system had a high sensitivity (94%) to detect severe jaundice, defined as total serum or plasma bilirubin >250 µmol/L, and maintained a high specificity (71%).

**Conclusions:**

Our smartphone-based system has a high potential as a tool for identifying NNJ. An iterative approach to product development, conducted by working on different tasks in parallel, resulted in a functional and successful product. By adhering to the requirements for regulatory approval from the beginning of the project, we were able to develop a market-ready mobile health solution.

## Introduction

### Background

Medical devices are a central part of medicine today and can be defined as any “products or equipment intended for a medical purpose” [1]. The development of novel medical devices is an area facing multiple barriers [2]. These include time for development, strict regulatory requirements, and financial aspects along with unknown market potential, limiting the number of devices reaching the clinical setting. Furthermore, numerous devices fail to scale up [3]. For the development of medical devices for infants and children, the challenges are even larger [4,5].

The field of eHealth and mobile health (mHealth) services has been rapidly developing, with technological advances opening new avenues for delivering health care, including point-of-care services. The expectations of this field have been high, especially in traditionally underserved areas [6]. Although many mHealth developments have shown promise, a key obstacle has been extending innovations from pilot projects and ideas to clinical implementation, a problem described as *pilotitis* [7]. Several factors have been linked to this phenomenon, such as regulatory requirements, lack of user perspective and engagement during development, and an absence of sustainable financial models in place, resulting in short-lived software applications and devices not tailored to the actual needs of the target population and users [8-10].

Neonatal jaundice (NNJ) is a common condition among newborns because it affects 60% of all term newborns and 80% of all preterm newborns [11]. Neonatal hyperbilirubinemia (NHB) is one of the leading causes of readmission to the hospital after discharge [12]. Although most cases are self-limiting, the condition could lead to severe consequences such as brain damage, sensory defects, or even death if not detected and treated in a timely fashion, and globally NHB remains an important cause of neonatal mortality [13-15]. Most of the severe cases occur in low- and middle-income countries. In addition, shortened stays at maternity wards after delivery are shown to increase the risk of hospital readmission for NNJ, which was further exacerbated during the COVID-19 pandemic [16,17]. To reduce the adverse consequences of NHB, timely detection of infants with the condition is important [18]. NHB can be detected in multiple ways, but the currently available methods all have specific limitations. Visual assessment is shown to be unreliable; transcutaneous bilirubin (TcB) measurements are too expensive for many settings; and measurement of total serum or plasma bilirubin (TSB) requires training and an invasive blood draw, in addition to having the necessary equipment. There is clearly a pressing need for novel detection technologies [19-21].

### Development of a Smartphone-Based System

On the basis of the need for novel technologies for NNJ detection, we developed a smartphone-based system with the following properties:

1. It provides accurate results.
2. It has been approved as a medical device.
3. It is easy to use.
4. It is affordable.

To achieve this, we (1) explored methods that could be adapted to our system, (2) tested and evaluated prototypes, and (3) developed a final product for formal validation before submission for conformity assessment.

In this paper, we describe our complete developmental process: going from product idea to a Conformité Européenne (CE)–marked medical device, which we call the *Picterus Jaundice Pro*.

## Methods

### Project Overview

**Figure 1:**
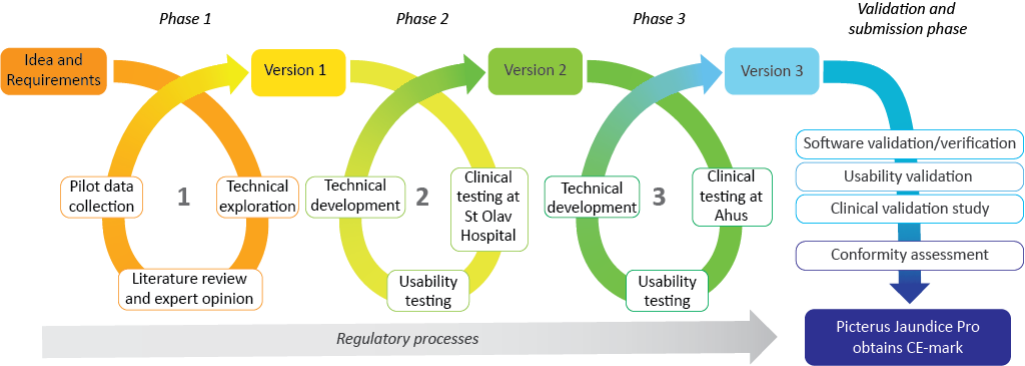
Overview of the development phases. Ahus: Akershus University Hospital; CE: Conformité Européenne.

We worked on this project from January 2014 to October 2021 in 3 different developmental phases before entering the final phase to obtain CE approval. We used an iterative approach that enabled us to work on different aspects of the product in parallel. The different stages included development of software and algorithms for bilirubin measurements, usability evaluations, product development, and collection of clinical data. An overview of the different stages is shown in Figure 1. All phases were carried out in compliance with the General Data Protection Regulation [22].

This project was initiated as a research project at the Norwegian University of Science and Technology and St Olav Hospital, both in Trondheim, Norway, and continued later in collaboration with the spin-off company Picterus AS.

### Phase 1

In the first developmental phase, we developed skin simulations to mimic the bio-optical properties of newborn skin and explored the technical properties of digital cameras used in smartphones [23].

The skin simulations were based on how light interacts with varying levels of the most important compounds that make up skin color, that is, melanin, blood, and bilirubin [24]. Each simulation resulted in a color. By running many simulations, we were able to generate a large database of bilirubin level–color pairs. We then compared the ability of different smartphone cameras to distinguish among colors in relation to different levels of bilirubin.

As color analysis of digital images depends on accurate color detection, we explored several ways of obtaining colors relevant for bilirubin detection. First, we tested a paper-based foldable spectrometer that was attached over the camera lens (Foldable Mini-Spectrometer; Public Lab) to separate the light into different wavelengths. Second, we attached band-pass filters (FB450-40 and FB550-40; Thorlabs Inc) over the camera lens that only allowed transmission of bilirubin-specific wavelengths. Finally, we obtained digital images using the standard camera on the smartphones of a fixed color reference, a MacBeth ColorChecker (X-Rite Inc). This ColorChecker is a reference card with different color samples with known properties and is commonly used by photographers to calibrate photographs [25].

A clinical pilot study was then performed to obtain sets of skin images of the chests of newborns. Thirty infants, dressed only in a diaper, were placed on their backs on a standard examination table, and 6 images were captured, 3 with flash and 3 without. A MacBeth ColorChecker was placed on the side of each infant’s chest. Images were taken at a distance of 40 cm from the chest using the built-in camera on a Samsung Galaxy S3 smartphone. TSB and TcB levels were measured for each infant within 60 minutes before or after image capture. The images were uploaded to a server and skin colors analyzed. The skin colors were first calibrated using the ColorChecker and then converted into bilirubin values based on our skin simulations. We then compared these values with the TSB values.

The results from the technical work and analyses of the newborns’ images were supplemented with input from clinical experience of jaundice assessment and literature searches on NHB. On the basis of published literature, we chose the best measurement site on newborns’ bodies to test our prototype system [26]. Version 1 of the system was then developed based on these findings (Figure 1).

### Regulatory Processes

We performed an initial assessment of whether a potential final product would be considered a medical device in accordance with European regulations [27,28]. On the basis of this assessment, a regulatory process for approval was initiated [29].

First, a quality management system in accordance with International Organization for Standardization 13485 standards was initiated. Thereafter, we conducted a comprehensive analysis of potential risks related to the product and created a risk management file. All risks related to the use of the product were evaluated and classified based on severity, risk of occurrence, and probability of intervention.

Definitions of the intended user and intended use as well as specifications of customer requirements were developed, which defined the criteria for technological development. All documentation of the product, including manufacturing, was incorporated into a technical file. We developed a clinical evaluation plan based on the relevant risks for the use of the product.

With regard to fulfilling regulatory requirements as a medical device, it should be noted that all the described regulatory processes do not have an end point but are continually updated throughout the lifetime of the medical device. The described regulatory documents in development phase 1 were continually updated during all phases of the project, and all involved participants were trained.

### Phase 2

In phase 2, we evaluated version 1 of the system.

#### Clinical Study

A clinical study was performed at St Olav Hospital, and relevant data were published in 2020 [30]. A cross-sectional study compared bilirubin levels from digital images, obtained with a smartphone together with a calibration card, with TcB and TSB levels. Four images from different distances were captured in a standardized setting. Infants were placed on a white cloth on an examination table under illumination provided by halogen light bulbs. All sunlight was blocked out with dark curtains.

#### Usability Testing

We used different methods to assess user experience of the system. First, a method known as guerrilla testing was applied [31]. This method provides rapid insights into how a system is perceived and involves little effort from test users. Nurses, midwives, and physicians at St Olav Hospital were asked to participate. This user group was chosen based on experience with handling newborns and knowledge of NHB. They were presented with version 1 of the system and asked to perform a supervised test on a baby doll. They were encouraged to express their opinions and experience while using the system.

Second, during the clinical study phase, research assistants who helped with data collection provided feedback on the system. This yielded additional insights because the research assistants used the system on live newborns and not on a doll. The images collected during the clinical study not only revealed technical issues but also provided insights into how the user captured the images.

#### Technological Development

Image sets with corresponding TSB levels from 34 randomly selected newborns in the clinical study were chosen and used to adjust the skin simulation models. We examined all collected images visually for quality and developed a method for an automated analysis of the image sets. Images that did not fulfill the quality criteria were excluded. To be included in the next phase of the analysis, the following criteria needed to be met: all color patches on the calibration card had to be clean and present in the image, there should be no major shadows over the skin or card, no object should have covered the lens, and the images should have been captured from the correct distance. For a set of images to be included, 3 out of the 4 images had to fulfill the quality criteria.

This procedure was then used to determine bilirubin levels in the remaining image sets (n=101). These levels were compared with TSB and TcB levels and evaluated using Pearson correlations and Bland-Altman plots.

A bench study was performed using version 1 of the system under different illumination conditions and with different smartphones. Skin phantoms were created by mixing gelatin with varying concentrations of bilirubin. Images of these phantoms were obtained under a standardized setting where the illumination could be altered. Four different smartphones were used, and 3 images for each phantom were obtained. The smartphones were placed on a stand to enable the capturing of stable images from the same distance and angle. A color calibration card, which was developed in phase 1, was used as a fixed reference for each image. The colors in the images before and after color calibration were compared, both under different light sources and among different smartphones.

The clinical study, bench testing, and usability studies resulted in an improved version of the system (version 2).

### Phase 3

In the third development phase (Figure 1), we tested and evaluated version 2 of the system and developed the final product.

#### Clinical Testing

Version 2 of the system was evaluated in a clinical study at the Akershus University Hospital, Lørenskog, Norway, and relevant data have been published [30]. We used inclusion criteria and methods that were similar to those used in the phase 2 clinical study. However, the illumination was not standardized, and images were captured under ambient light conditions to evaluate the performance in settings closer to those in which the final product would be used.

#### Usability Testing

Usability testing in phase 3 was also conducted by applying the guerrilla method using a doll as well as by recording the experiences of research assistants taking part in data collection.

#### Technological Development

Images collected in the clinical study of phase 2 were used for evaluating the clinical performance of the system, as well as for developing a method for automated quality checks of the images. Moreover, a method for checking the quality of the calibration card was developed. We set up the server to perform automated bilirubin measurements from approved image sets, and we developed a communication module that would report the result back to the app on the user’s smartphone.

To ensure safe storage and transport of the cards, we designed special packaging for the calibration cards in compliance with regulatory requirements. Instructions for use were provided in both paper and web-based formats, as well as an in-app teaching module. The results from usability testing were used to improve the design of the calibration card as well as the smartphone app.

The combined findings in the third phase of the development led to version 3 of the system.

### Validation and Submission Phase

In the last phase, we aimed to finalize a fully tested system that was ready for certification as a medical device. To achieve this, we performed validation and verification tests of the software and hardware, as well as formal usability testing, and executed a clinical validation study of version 3 of the system.

#### Software and Hardware Validation and Verification

We developed validation and verification tests of the software and hardware of version 3 to prove that the system meets user needs and intended use criteria, as well as system requirements and specifications [32]. These tests are based on the needs and requirements of users, as well as risks identified in the risk assessment.

#### Usability Validation

To be in accordance with regulatory requirements, a formal usability study was performed. Sixteen health care workers were recruited and shown the system with the app, instructions for use, and calibration card. The risk analysis identified potential misuse of the product, and the users were presented with 10 different test cases that simulated scenarios where such misuse could happen. Tests were performed on a baby doll lying on an examination table. The test cases included basic tests to assess the ease of downloading and installing the app, as well as the users’ understanding of the intended use of the product. Specific pass or fail criteria for each test case were defined in advance.

#### Clinical Validation Study

##### Study Design

We conducted a cross-sectional prospective study in the maternity ward and a breastfeeding outpatient clinic at St Olav Hospital. Data collection took place from September 5, 2019, to September 9, 2020.

##### Study Participants

Newborns were selected for the study if they were aged 1 to 15 days, born at term (37 weeks of gestation or more), and with birth weights ranging from 2500 g to 4500 g. Newborn infants with signs of disease or skin conditions other than jaundice and who received advanced medical treatment or phototherapy were excluded.

##### Recruitment

To include newborns with a wide range of bilirubin levels, we recruited them from 3 different groups: newborns at the maternity ward who needed a TSB determination for clinical purposes, newborns without visible jaundice but whose parents were willing to let them undergo a TSB sample drawn during their newborn screening when they were aged approximately 48 hours, and newborns brought to the breastfeeding clinic for follow-up care and who needed TSB determination for clinical purposes. The parents were asked to participate in the study, and informed written consent was obtained.

##### Data Collection

For each newborn, the following variables were collected: day and time of birth, birth weight, gestational age, sex, and feeding type, as well as mother and newborn blood type and Rh type. A blood volume of 600 µL was obtained by heel prick or venipuncture and stored in light-protected containers. The blood samples were analyzed at St Olav Hospital by applying the vanadate oxidation method using the Advia Chemistry XPT system (Siemens Healthcare GmbH). Within 60 minutes of blood collection, each newborn was placed on an examination table, and a measurement using the Picterus Jaundice Pro was performed. A color calibration card was placed over the sternum, and an image set, consisting of 3 images with flash and 3 without, was collected using a Samsung Galaxy S7 smartphone with version 3 of the Picterus Jaundice Pro. For each image, color correction was carried out and bilirubin levels determined. Bilirubin assessment using a TcB-measuring device (Jaundice Meter JM-105; Dräger) was performed immediately thereafter. All measurements were performed by the same trained person following hospital routines and instructions from the manufacturer.

##### Statistical Analysis

Bilirubin measurements using the different methods were analyzed using the Kruskal-Wallis test with the Dunn multiple comparison post hoc test. The correlations between Picterus Jaundice Pro bilirubin levels and TSB levels were evaluated using Pearson *r*. Bland-Altman plots were performed to determine the mean bias and 95% CIs between Picterus Jaundice Pro and TSB levels. Receiver operating characteristic curves were constructed to determine the sensitivity and specificity to detect severe jaundice, defined as TSB >250 µmol/L (14.6 mg/dL). Analyses were performed using MedCalc (version 20.109; MedCalc Software Ltd).

#### Submission for Conformity Assessment

A notified body was contracted to perform the conformity assessment and collect all documents to comply with Medical Device Regulation (MDR) approval. The documents included the technical file describing all components of the product, a clinical evaluation report, a plan for postmarket surveillance and clinical follow-up to capture user feedback and confirm safety of the product, the risk analysis, and the quality management system.

### Ethics Approval and Study Registration

The clinical studies were approved by the Regional Committee for Medical and Health Research Ethics of Southeast Norway (2014/618) and by data protection officers at both St Olav Hospital and Akershus University Hospital. The validation study was approved by the Norwegian Medicines Agency as a study of a non–CE-marked medical device. The clinical studies were registered in ClinicalTrials.gov (NCT03007563 and NCT04182555).

## Results

**Figure 2:**
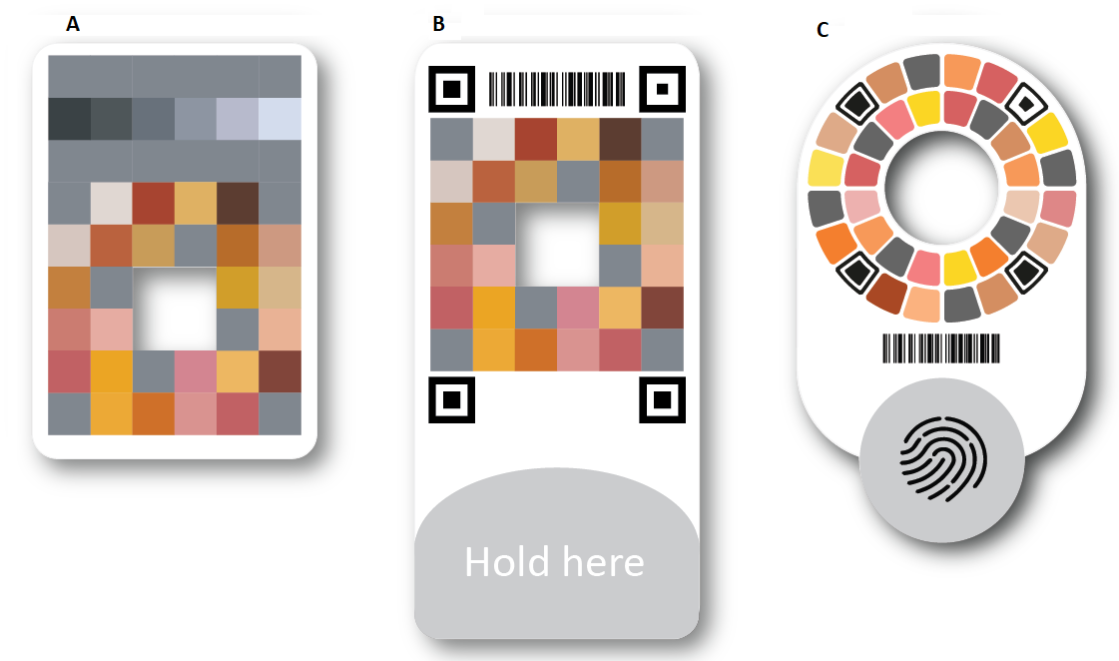
Versions of the calibration card. (A) Phase 1. (B) Phase 2. (C) Phase 3 (final version).

**Figure 3:**
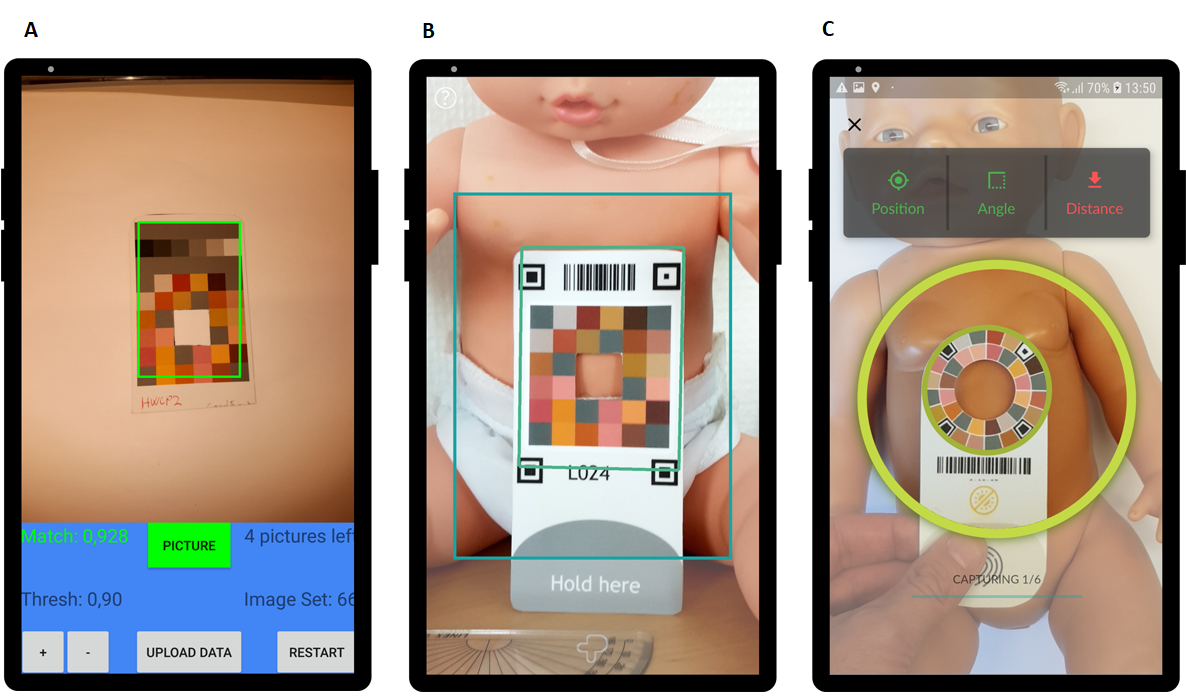
Versions of the app slides of the image capture module. (A) Phase 1. (B) Phase 2. (C) Phase 3 (final version).

The development of the Picterus Jaundice Pro involved 3 phases before the final version was approved for CE certification as a medical device (Figure 1). The 3 phases produced 3 iterations of the calibration card (Figure 2) and 3 iterations of the image capture interface of the app (Figure 3).

### Phase 1

#### System With 3 Major Components

In phase 1, we developed a system consisting of 3 major components: an app to collect and upload images of the skin of newborns, functionality to accurately calibrate the colors in the images, and a server that could communicate with the smartphone and where images could be stored and analyzed.

Initial assessment of the smartphone cameras revealed that they were able to distinguish among colors reflecting different levels of bilirubin [23]. However, under varying illumination conditions, using a fixed reference in the images in the form of the color calibration card was the only way to detect colors with the needed accuracy; accordingly, other methods of color detection were abandoned. This resulted in a system consisting of a color calibration card, an app, and a server.

In the pilot testing in phase 1, the newborns had a mean TSB level of 166 (SD 74) µmol/L (mean 9.71, SD 4.33, mg/dL) and a mean TcB level of 150 (SD 120) µmol/L (mean 8.78, SD 7.02, mg/dL). The bilirubin measurements from the images had a mean level of 190 (SD 119) µmol/L (mean 11.1, SD 6.96, mg/dL). The TSB and TcB levels were highly and significantly correlated (Pearson *r*=0.92; *P*=.005). Comparison of bilirubin values from the images with TSB and TcB levels revealed no significant correlation (Pearson *r*=0.04; *P*=.23).

On the basis of these findings, we identified several areas for improvement, with *how* images were captured being most essential. The design of the ColorChecker [25] was not optimal for color calibration of images of newborns’ skin, and the ColorChecker needed to be positioned closer to the measurement site.

As a result, we developed the first version of the color calibration card (Figure 2A). This card consisted of various color and gray patches. To get these patches close to the measuring site, the card had a hole in the center showing where to perform skin measurements. We implemented a spectral printing method, which makes colors consistent when exposed to different illuminations [33]. We printed the cards using an inkjet printer and standard photo paper (HP DesignJet Z7300 printer and HP Premium Matte Photo Paper; HP Inc), which made it possible to print these cards at a low price. To ensure color consistency, all cards were measured with a spectrophotometer after printing.

Although image standardization with regard to distances and angles among the newborns was attempted, the built-in camera app of the smartphones was not adequate for this purpose. We therefore developed an app with an image capture module (Figure 3A) to standardize how images were obtained. The app was developed for an Android operating system. We included functionality to store and upload images. In addition, we set up a server that could communicate with the app and link image sets to unique identity numbers for each newborn.

All implemented features of version 1 of the app, color calibration card, and server are described in Textbox 1. The color calibration card of version 1 is shown in Figure 2A, and the app slide of the image capture module is presented in Figure 3A.

**Description of version 1.**

- App
  Functionality to take sufficient images for clinical study
  Communicates with server to upload images
  Software for recognizing card
  Takes images from different distances
  Takes images with and without flash
  Image sets coded to unique ID
  Indicates to user when to take photographs
  Indicates to user how to position smartphone
  Crops image
- Calibration card
  24 color and 8 gray patches
  Strip of 6 different gray tones
  Hole for skin analysis
  Colors close to hole
  Opaque
  Matte surface to reduce reflections
  Size that fits on infant’s chest
  Safe for the newborn: biocompatible material and no sharp edges
- Server
  Receives and stores images with unique ID for later analysis

#### Regulatory Compliance

The initial assessment of the final system concluded that the system could be considered class 1 medical device software according to the Medical Device Directive [27]. Through the timeline of the development, European regulations regarding medical devices changed, with the MDR replacing the Medical Device Directive [28]. A new assessment of the final system according to the updated regulations classified the system as a class 2a medical device. The color calibration card had been classified as a class 1 medical device accessory, and this classification remained the same after the change to the MDR.

The intended users of the system were defined as health care personnel, with intended use at hospitals, clinics, health stations, or during home visits. The system was identified as a screening system that assists health care workers in their assessment of NNJ and not as a stand-alone diagnostic tool. According to MDR specifications, the device was to be used on newborns born at term, with normal birth weight, and not showing signs of pathologic jaundice.

During the risk assessment, we found 3 risks that needed to be mitigated by a clinical evaluation: confirmation of a relationship between bilirubin levels in the skin and in blood, verification that bilirubin measurements in digital images in general correlate to bilirubin levels in blood, and demonstration to prove that bilirubin measurements using our system are correlated to bilirubin levels in blood. The first and second risks were resolved by reviewing published papers, but for the third risk we had to gather our own clinical data to have the system approved as a medical device.

To be compliant with the General Data Protection Regulation, all images were cropped so that no identifiable features of the newborns were visible.

### Phase 2

In phase 2, we tested, evaluated, and improved upon the first prototype of the system (version 1).

During the clinical study of phase 2, a total of 181 newborns were recruited: 36 (19.9%) had image sets that had technical errors or were completely missing, another 10 (5.5%) had missing blood samples or were excluded because of miscoding or not meeting our inclusion criteria, and a subset of 34 (18.8%) newborns who were used to calibrate our model were also excluded from further analysis, leaving 101 (55.8%) image sets available for final analysis. Bilirubin values from the images were highly and significantly correlated to TSB values (Pearson *r*=0.83; *P*<.001) and TcB values (Pearson *r*=0.85; *P*<.001) [30].

An analysis of the image sets with technical errors revealed several weaknesses with version 1, and usability testing showed that the method of obtaining images was inadequate. The technical errors in the 36 rejected image sets included images where the infants’ clothes covered portions of the color calibration card or the fingers of the researcher covered the lens, as well as image sets where the color calibration card or the infant was not captured in the image.

The software for recognizing the calibration card did not perform as intended, and there was an unwanted delay between the time that the app judged that conditions for capturing an image were met and the time that the actual images could be obtained. After testing different methods, we added 4 QR position codes to the calibration card and implemented updated software to recognize these position codes (Figure 2B). The position codes served as distinct and easily recognizable features on the card and made sampling of the color patches from the images more stable and easier to automate.

In version 1, the user had to press a button to capture images, which made it difficult to hold the smartphone steady. We implemented automated image capturing and added a camera shutter sound as well as a progress bar indicating the progress of the image capture operation.

Images from different distances did not improve correlations, added complexity, and reduced usability; therefore, a standard distance of 25 cm was chosen. Testing on live newborns showed that it was challenging to have the color calibration card stay in place during image capture, and we increased the size of the card, adding a section where the user could hold the card without affecting the color patches or the QR position codes (Figure 2B).

Analysis of the images of the skin phantoms showed that the method of color calibration was able to correct for changes in illumination as well as differences among the cameras in the 4 different smartphones that we tested. The analysis further showed that some of the patches on the calibration card were not needed and were therefore removed (Figure 2B).

All implemented features in version 2 of the app, color calibration card, and server are described in Textbox 2. The color calibration card of version 2 is shown in Figure 2B, and the image capture interface is presented in Figure 3B. The implementation of the combined findings from the second phase led to version 2 of the system with an updated interface for the app and a new version of the color calibration card.

**Description of version 2.**

- App
  Updated software for recognizing calibration card
  Takes images from fixed distance
  Takes images from same angle
  Takes images automatically
  Only takes images when conditions are met
  Plays shutter sound when taking images
  Displays a frame of the calibration card to ease positioning of the phone
  Illustrates progress of image capture operation
  Collects data on gestational age, birth weight, and current age
- Calibration card
  Flap to hold the card
  Barcode to identify unique card
  QR position codes to improve card detection
- Server
  Receives and stores images with unique ID for later analysis

### Phase 3

In phase 3, we tested and evaluated version 2 and developed a final product (version 3) that was ready for formal validation before submission for conformity assessment.

During the clinical study of phase 3, a total of 161 newborns were recruited, of whom 3 (1.9%) had been miscoded and were excluded. Updating of the software and calibration card resulted in improved image capture quality: no image set had to be excluded. We determined a bilirubin value in the remaining 158 infants, of whom 84 (53.2%) had a TSB test performed, and the bilirubin measurements from our system were highly and significantly correlated to the TSB values (Pearson *r*=0.85; *P*<.001) and TcB values (Pearson *r*=0.79; *P*<.001) [30].

**Figure 4:**
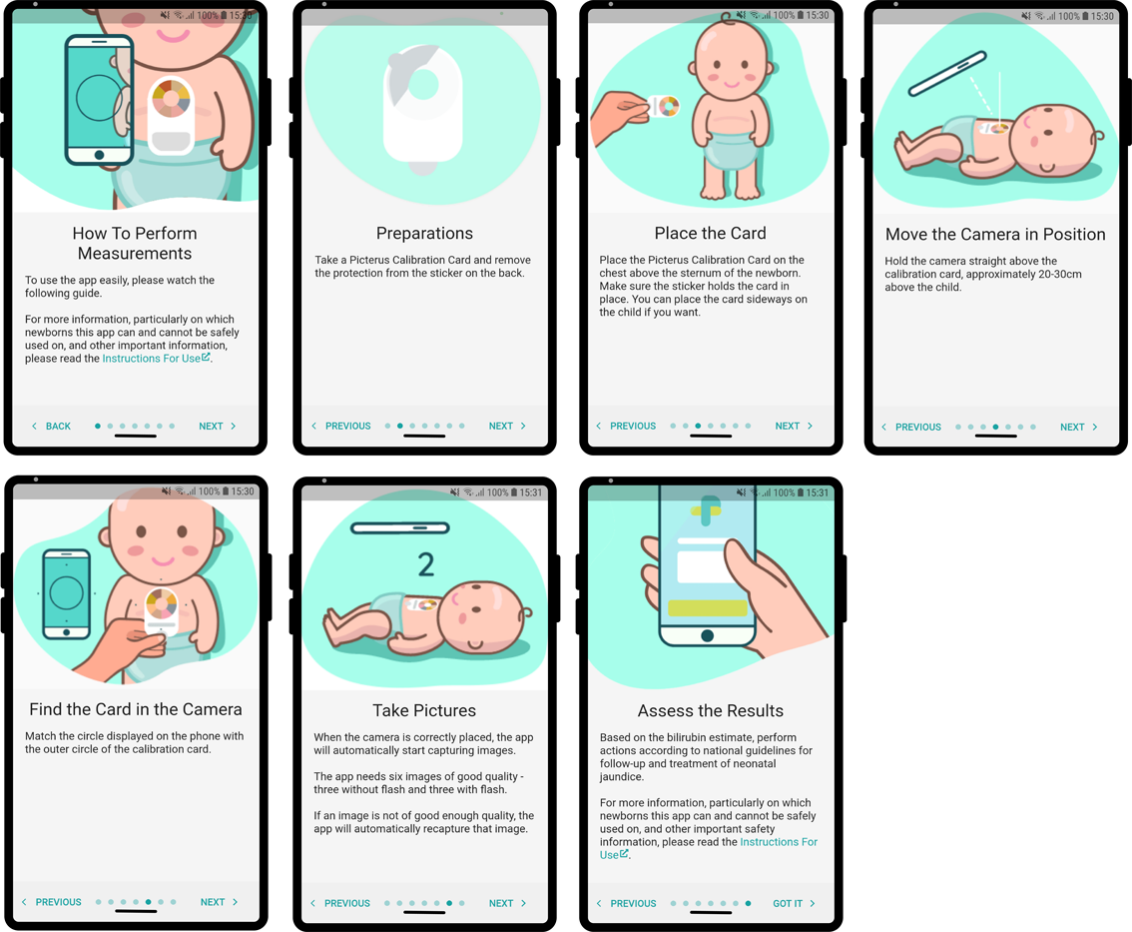
App slides of the training module.

Usability tests and the experience of the research assistants showed that the image capture process was still not optimal, and it was difficult to align the smartphone and the card. We changed the design of the calibration card to a circular layout that reduced the need for this alignment and changed the user interface accordingly (Figures 2C and 3C). Another challenge was that users had to hold the card in place with one hand and then position the smartphone with the other. This was especially difficult when the newborn was moving. Therefore, we added double-sided medical tape, intended for use on newborn skin, on the back of the card to hold it in place while capturing images. To help new users, we developed a training module and implemented this in the app (Figure 4).

On the basis of the outcomes of the clinical study, we developed multiple quality checks for both the calibration card and the images. Inputs to these quality checks were based on continually updated risk analysis. The quality checks for the calibration card included tests to ensure that there were no shadows over the card, the card was not manipulated, nothing was spilled on the card, and the card was not damaged in other ways. The quality checks of the skin patch included checks to determine that the test area only captured human skin, that the skin had no rash, and that there were no shadows in the area chosen for analysis.

To maintain the colors on the calibration card, we designed special packaging that would protect the card from exposure to sunlight. Finally, instructions for use, terms and conditions, and a privacy policy were developed in accordance with relevant requirements.

All implemented features of version 3 of the app, the color calibration card, and the server are described in Textbox 3. The color calibration card of version 3 is shown in Figure 2C, and the image capture interface is presented in Figure 3C.

**Description of version 3.**

- App
  Gives immediate result
  On-boarding sequence
  Learning module
  Shows terms and conditions
  Instructions for use
  Stores latest measurement results
  Reduces need for user input
  Shows user whether position, angle, and distance are within required levels
  Developed using software that would enable use of both Android and iOS smartphones
  Includes functionality to translate app text into multiple languages
- Calibration card
  Safe packaging of card
  Circular design to ease alignment with the smartphone
  Sticker to hold the card in place on the chest
  Increased size of hole for skin analysis
  Separate version for multiple use and possible to sanitize card
  Multiple-use card (laminated)
- Server
  Performs automated bilirubin assessment from images
  Communicates result to app
  Rejects image sets if quality requirements are not met
  Calibration card present in the image
  All color patches (colors and gray) on the calibration card present
  Barcode present in all images
  Colors in the card hole for the skin within normal range for skin colors (meaning no object other than human skin is being evaluated)
  Cross validation of colors
  Checks whether card is damaged

### Validation and Submission Phase

In the final phase, we demonstrated that the system was ready for approval as a medical device. Using version 3, we performed validation and verification tests of the software and hardware and also conducted formal usability testing and a clinical validation study.

#### Software and Hardware Validation and Verification

Validation and verification tests of version 2 were developed and executed, and the system passed all the tests.

#### Usability Validation

During formal usability testing, one of the test participants failed in 1 of the 10 test cases regarding the definition of the intended users of the system. This was not considered critical, and no risk mitigation was required.

#### Results of Clinical Validation Study

A total of 248 newborns were recruited in the study, of whom 201 (81%) met the inclusion criteria for the intended use of the system and were included for data analysis. Birth weight, gestational age, and TSB and TcB levels as well as bilirubin measurements from the Picterus Jaundice Pro system are shown in Table 1. Thirty-four newborns were classified with severe hyperbilirubinemia, defined as TSB >250 µmol/L (14.6 mg/dL).

**Figure 5:**
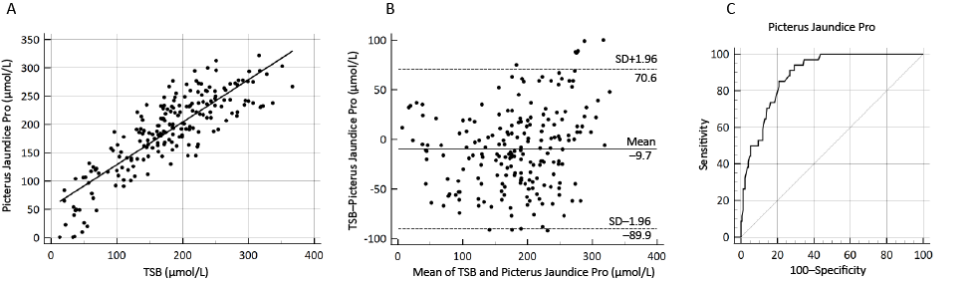
Correlation between Picterus Jaundice Pro and total serum bilirubin (TSB) levels illustrated in (A) a scatterplot, (B) a Bland-Altman plot, and (C) a receiver operating characteristic curve.

The scatterplot (Figure 5A) shows a significant positive correlation between TSB and Picterus Jaundice Pro values (Pearson *r*=0.85; *P*<.001). Correlations between TcB and Picterus Jaundice Pro values showed similar results (Pearson *r*=0.85; *P*<.001). The mean difference between Picterus Jaundice Pro values and TSB levels was –9.7 µmol/L (95% CI –89.9 to 70.6 µmol/L; –0.57 mg/dL, 95% CI –5.26 to 4.13 mg/dL). No systematic overestimations or underestimations of bilirubin levels were observed (Figure 5B). Receiver operating characteristic curve (Figure 5C) analysis of data from newborns with severe hyperbilirubinemia, defined as TSB >250 µmol/L (14.6 mg/dL), showed an area under the curve value of 0.89 and a Youden index with cutoff at >214 µmol/L (12.5 mg/dL), resulting in a sensitivity of 94.1% and a specificity of 70.7%.

**Table 1:** Clinical characteristics of the included newborns (N=201).

Characteristics				Values, mean (SD; range)
Birth weight (g)				3642 (460; 2510-4830)
Gestational age (weeks)				39.7 (1.4; 37-42)
**Bilirubin levels**				
	**µmol/L**			
		Picterus Jaundice Pro	186.6 (66.5; 1-322)	
		TSB^a,b^	178.2 (76.5; 13-367)	
		TcB^c,d^	156.7 (70.4; 0-304)^e^	
	**mg/dL**			
		Picterus Jaundice Pro	10.9 (3.9; .1-18.8)	
		TSB^b^	10.4 (4.5; 0.8-21.5)	
		TcB^d^	9.2 (4.1; 0-17.8)^e^	

^a^TSB: total serum bilirubin.

^b^Total serum bilirubin and transcutaneous bilirubin levels and bilirubin measurements from the Picterus Jaundice Pro system were analyzed with the Kruskal-Wallis test with the Dunn multiple comparison post hoc test.

^c^TcB: transcutaneous bilirubin.

^d^Information from 2 newborns is missing.

^e^*P*<.05 versus total serum bilirubin.

#### Submission for Conformity Assessment

On the basis of the risks outlined in the results of phase 1, a literature review was performed by searching PubMed, MEDLINE (via Ovid), and Scopus databases. Relevant information from 59 articles was included in the review.

Our own clinical data were insufficient to claim that the device could be used on newborns born to parents with the darkest skin types, defined as type 5 and 6 on the Fitzpatrick scale, and this limitation was added to the intended use of the product. A notified body reviewed all documentation of our system and performed 2 on-site audits before the certificate of the CE mark for the device was issued on October 22, 2021 [34]. For the calibration card, which was classified as an accessory, a self-declaration was sufficient to obtain the CE mark.

## Discussion

### Summary

In this paper, we have described the development of a new digital system for screening of NNJ using a CE-certified medical device. By working in parallel along technical, clinical, usability, and regulatory axes, we developed iterations of the system that progressively resulted in a medical device that was approved as a CE-marked medical device. Clinical validation studies demonstrated that the Picterus Jaundice Pro bilirubin measurements highly correlated with TSB levels with a high sensitivity to identify newborns with severe hyperbilirubinemia.

For the development of this system, we set 4 objectives:

- First, we wanted to create a device that provides accurate results. The clinical validation study demonstrated a strong correlation between Picterus Jaundice Pro and TSB measurements. This objective was, however, only partially met because validation for all skin types was not yet demonstrated.
- Second, the system had to be approved as a medical device, and this was achieved by obtaining the CE mark. The CE mark is only valid in the region where this mark is accepted; however, this proves that our system will fulfill safety requirements, and adaptation to regulatory requirements in other regions will be easier.
- Third, the system should be easy to use. This is a necessity for lowering the threshold for using the system and making it accessible even for users with limited experience in using digital tools. Through usability testing and iterative development, we received feedback on different versions of the system and gradually improved it to meet the needs of the intended users.
- Fourth, we aimed to create a system that would be affordable even in low-income settings. A key advantage of digital solutions is the relatively low costs of scaling up the solution. However, maintenance costs will still apply as will costs involved in product approval. Furthermore, regulatory requirements result in continuous costs incurred on safety and clinical performance follow-up. The costs of production of the calibration card are low, but distribution costs will increase the cost for the user. Nonetheless, compared with other relevant medical devices for NHB, the total costs are substantially lower.

### Strengths

From the initial phase of the project onward, the overarching goal was to convert our work into actual improvement in newborns’ health by creating a product ready for users and also to develop the technology. The iterative approach combining usability and clinical testing in parallel with technological development enabled us to gradually develop a product that fulfilled our objectives. The guerrilla testing method is easy to conduct and made it possible to reach the intended users despite their busy clinical work.

Regulatory compliance work has been a substantial part of this development and included tasks that are often seen as cumbersome, expensive, and challenging. The rationale behind these processes is to prevent unsafe devices from reaching patients and to ensure that the benefits of a device can justify any remaining risks related to it. In this development, we identified potential risks from the beginning and evaluated and mitigated the risks when needed. Implementing the regulatory processes from the beginning ensured feasibility of the actual product in terms of increased patient safety, which will lead to increased acceptance of the product by users.

Compared with projects that are a result of technology discoveries, an advantage of this project is that it originates from a clinical need. This factor, often called “the why,” has been highlighted as key to scaling and spreading innovations [3].

### Limitations

Although we developed, user-tested, validated, and obtained regulatory approval for a novel system fulfilling a clinical need, further development is needed to ensure successful implementation of the innovation [3,35].

Although we performed usability testing of the device, at this stage we were not able to test the system in clinical practice. We need to understand how this system will fit into the daily workflow of delivery of health care and how users will experience added value [10]. Use in clinical practice would further contribute to understanding which other features the system should include; for instance, the system currently provides no interpretation of the results, and this could be an important feature for further development. The largest burden of NHB today is experienced in low- and middle-income countries, and a limitation of this project is that we did not perform usability testing with users in such settings. Although the usability experience regarding the image capture module is likely to be similar, implementation of new features must be adapted to specific contexts, especially because many newborns in low-resource settings are seen by health care workers with less education.

Moreover, the ability to scale up the innovation is required to ensure successful implementation. The system was approved and tested for a limited set of smartphones, but to scale up the system, it must work and be approved for use on most smartphones. The calibration cards are currently being printed on a small scale, and a system for mass production needs to be developed. The system further requires connectivity, and for remote and less developed areas this would be a limiting factor. As sub-Saharan Africa and South Asia are among the regions experiencing the largest burden of NNJ, it will be important to have the system approved for newborns with all skin pigmentations.

Finally, sustainability of innovations includes financial sustainability, with appropriate business models generating revenue. Development of such models has been part of our work but is not included in this paper. In general, the organization of a health care system is complex and differs from country to country, and our business models will need to be adjusted depending upon how health services are financed within each specific market.

### Conclusions

In conclusion, we have described the development of a novel system for screening of NNJ from conceptualization to a CE-certified medical device. By following an iterative approach and by working along different axes in parallel and with considerations given to requirements for regulatory approval from the beginning, we developed a market-ready mHealth solution.

## Abbreviations

CE: Conformité Européenne
MDR: Medical Device Regulation
mHealth: mobile health
NHB: neonatal hyperbilirubinemia
NNJ: neonatal jaundice
TcB: transcutaneous bilirubin
TSB: total serum bilirubin
